# Moyamoya disease susceptibility gene *RNF213* links inflammatory and angiogenic signals in endothelial cells

**DOI:** 10.1038/srep13191

**Published:** 2015-08-17

**Authors:** Kazuhiro Ohkubo, Yasunari Sakai, Hirosuke Inoue, Satoshi Akamine, Yoshito Ishizaki, Yuki Matsushita, Masafumi Sanefuji, Hiroyuki Torisu, Kenji Ihara, Marco Sardiello, Toshiro Hara

**Affiliations:** 1 Department of Pediatrics, Graduate School of Medical Sciences, Kyushu University, Fukuoka 812-8582, Japan; 2 Department of Pediatrics, Faculty of Medicine, Oita University, Yufu 879-5593, Japan; 3 Section of Pediatrics, Department of Medicine, Fukuoka Dental College, Fukuoka 814-0193, Japan; 4 Department of Molecular and Human Genetics, Baylor College of Medicine, Jan and Dan Duncan Neurological Research Institute, Texas Children’s Hospital, Houston 77030, USA

## Abstract

Moyamoya disease (MMD) is a cerebrovascular disorder characterized by occlusive lesions of the circle of Willis. To date, both environmental and genetic factors have been implicated for pathogenesis of MMD. Allelic variations in *RNF213* are known to confer the risk of MMD; however, functional roles of RNF213 remain to be largely elusive. We herein report that pro-inflammatory cytokines, IFNG and TNFA, synergistically activated transcription of *RNF213* both *in vitro* and *in vivo*. Using various chemical inhibitors, we found that AKT and PKR pathways contributed to the transcriptional activation of *RNF213*. Transcriptome-wide analysis and subsequent validation with quantitative PCR supported that endogenous expression of cell cycle-promoting genes were significantly decreased with knockdown of *RNF213* in cultured endothelial cells. Consistently, these cells showed less proliferative and less angiogenic profiles. Chemical inhibitors for AKT (LY294002) and PKR (C16) disrupted their angiogenic potentials, suggesting that RNF213 and its upstream pathways cooperatively organize the process of angiogenesis. Furthermore, RNF213 down-regulated expressions of matrix metalloproteases in endothelial cells, but not in fibroblasts or other cell types. Altogether, our data illustrate that RNF213 plays unique roles in endothelial cells for proper gene expressions in response to inflammatory signals from environments.

Moyamoya disease (MMD) represents a specific intracranial vascular disorder characterized by progressive, occlusive lesions of internal carotid arteries and branches in the circle of Willis^1^,^2^. To compensate the decreased blood flow in the affected brain area, the fine vascular network of *Moyamoya*, a Japanese word meaning “puffs of smoke”, develops as arterial stenosis progress^1^,^2^. Earlier studies demonstrated that environmental factors including varicella zoster virus infection contributed to the development of MMD^3^,^4^. On the other hand, population-based studies pointed to the higher incidence of MMD in oriental populations than those in Caucasians, suggesting that certain genetic backgrounds may also confer the risk for the development of the vascular lesions^5^.

Genetic studies for MMD patients have been conducted to identify candidate disease susceptibility loci^6^,^7^,^8^,^9^,^10^. Notably, several groups have demonstrated that single nucleotide variations in the *RNF213* gene had a strong association with the onset of MMD in both familial and sporadic cases^11^,^12^. The human *RNF213* gene encompasses a 137,922-bp region at chromosome 17q25.3 (chr17:78,234,660–78,372,581) and consists of 68 exons with 67 protein-coding exons. The encoded 596-kDa protein, RNF213, harbors AAA-type ATPase, alpha-2-macroglobulin, and ring finger domains from its amino to carboxyl terminus^13^. Because of the presence of ring finger domain(s), RNF213 is considered a member of E3 ubiquitin ligase protein family. Recently, RNF213 has been reported to be associated with angiogenesis^14^; however, little is known about its endogenous functions or its pathogenic roles in MMD^13^,^15^.

To uncover the functional roles of RNF213 and pathogenic processes underlying MMD, we took advantage of bioinformatics approaches to analyze hundreds of transcriptomic data publicly available at open databases^16^. The bioinformatics data predicted that RNF213 might act cooperatively with other molecules under inflammatory signals. Based on this unbiased prediction, we investigated whether RNF213 might respond to pro-inflammatory stresses. Through a series of functional studies, we herein propose that RNF213 links the gap between environmental risk factors for the onset of MMD and endogenous signaling that is essential for angiogenesis.

## Results

### RNF213 is associated with immune response

We reasoned that identifying endogenous functions of RNF213 would facilitate our work towards unraveling the pathogenic mechanisms of MMD. To this end, we hypothesized that co-expression analysis can drive the prediction of functional pathways that RNF213 might regulate or be involved in. We took a bioinformatics approach to perform an unbiased analysis on the expression profile of *RNF213* in a large collection of human tissues and experimental conditions^16^,^17^. Gene Ontology (GO) analysis of the genes that showed highly correlated in expressions with *RNF213* was then performed to infer putative pathways where RNF213 might play a functional role (Supplementary Fig. S1 and Supplementary Table S1). We found that the GO categories of “immune response”, “response to virus”, “defense response”, “inflammatory response”, and “innate immune response” were significantly enriched and were consistently ranked at the top list of GO categories (Supplementary Fig. S1 and Supplementary Table S1). These data suggested that *RNF213* may be functionally associated with immune systems and/or virus defense. It was also noted that the GO term of “protein kinase cascade” was significantly enriched in the co-expression analysis. *RNF213* was therefore likely co-regulated with other genes under stressed conditions, such as inflammation or infections.

### Pro-inflammatory cytokines activate the transcription of *RNF213*.

**Figure 1:**
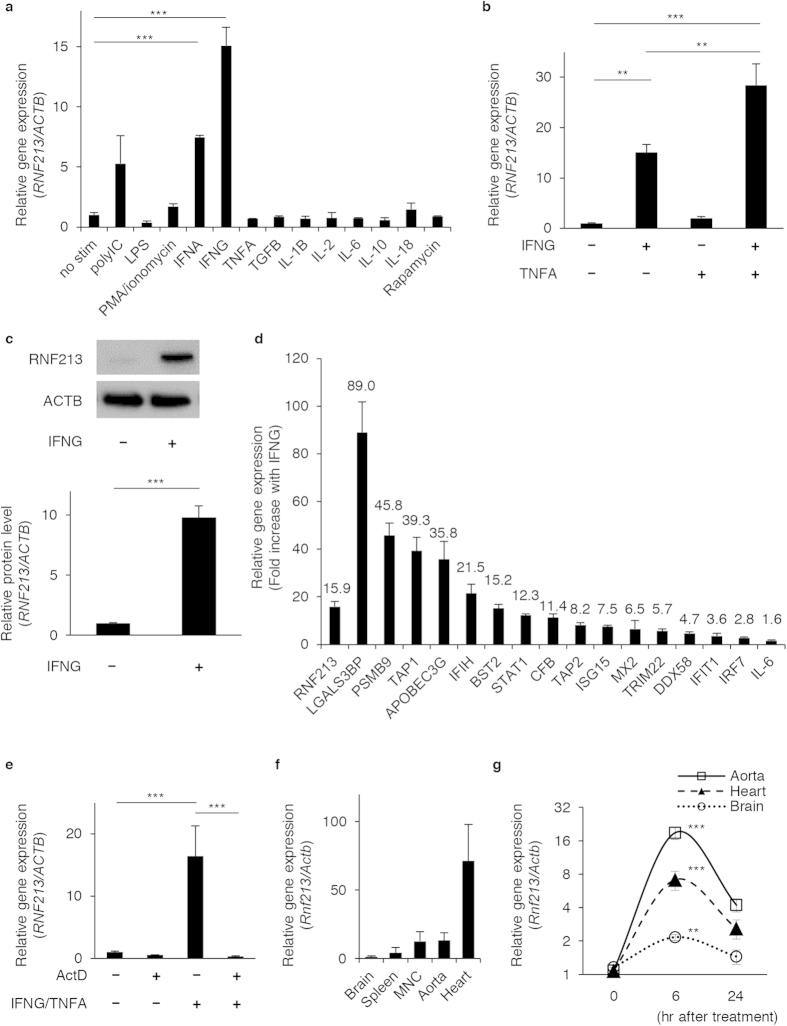
*RNF213* is transcriptionally activated by IFNG and TNFA *in vitro* and *in vivo.* (**a**) Relative expressions of *RNF213* in HUVECs when stimulated with various ligands for innate immunity and cytokines in comparison to that of control (“No Stim”). (**b**) Synergistic effects of IFNG and TNFA treatments on *RNF213* expression in HUVECs. (**c**) Western blot analysis for the RNF213 protein induction with IFNG treatments in HUVECs. Quantified results are plotted under the blotting image. (**d**) Coinstantaneous inductions of *RNF213* and other co-expressed genes upon IFNG treatments of HUVECs. (**e**) Suppression of *RNF213* induction after IFNG and TNFA treatments by actinomycin D (ActD) for HUVECs. (**f**) The steady-state *Rnf213* expressions in various tissues of female mice at 4-weeks of age (compared with Brain). (**g**) Acute induction of *Rnf213* transcripts after intraperitoneal injections of IFNG and TNFA *in vivo*. (**a–g**) Data are shown as mean ± SD values from 3 or more independent assays and analyzed using Dunnett’s test (**a**,**g**) Tukey’s HSD test (**b**,**e**) and Student’s t-test (**c**). **p < 0.01, ***p < 0.001.

Based on the bioinformatic prediction above, we next explored the exogenous ligands that may affect the endogenous expression of *RNF213* in cultured endothelial cells. We first stimulated HUVECs with various ligands for innate immunity or cytokines, including polyI:C, LPS, PMA/ionomycin, IFNA, IFNG, TNFA, TGFB, IL-1B, IL-2, IL-6, IL-18, and rapamycin^18^. We found that *RNF213* mRNA in HUVECs was significantly up-regulated when the cells were treated with IFNA or IFNG (Fig. 1a). Because TNFA was known to promote angiogenesis^19^,^20^, we additionally examined *RNF213* mRNA level with co-stimulation of the cells with TNFA and IFNG. The result showed that TNFA and IFNG combination further enhanced the expression level of *RNF213*, supporting the synergistic effects of pro-inflammatory cytokines on endothelial gene responses (Fig. 1b). Similar results were also obtained in HCAECs (Supplementary Fig. S2). The stimulatory effect of IFNG on the expression of *RNF213* in endothelial cells was verified at the protein level (Fig. 1c). We also tested whether the genes that were predicted to be co-regulated with *RNF213* (Supplementary Table S1) were also up-regulated with such cytokine treatments. We randomly selected 15 genes (25.4%) from those listed in Supplementary Table S1, and appended *IL-6* as a positive control for the IFNG treatment^21^. We ensured that IL-6 expression was increased 1.6-fold to the basal level, and that all of the 15 genes were robustly induced by the IFNG treatment (Fig. 1d). We also verified that the increase of *RNF213* mRNA was the result of transcriptional activation, rather than increased stability of mRNA, because a low-dose treatment with the RNA polymerase inhibitor, actinomycin D (500 μg/ml), efficiently blocked the acute increase in the amount of *RNF213* transcripts upon cytokine stimulation (Fig. 1e). We therefore concluded that the expression of *RNF213* was up-regulated by pro-inflammatory cytokines in a transcription-dependent manner in cultured endothelial cells.

We then investigated the relevance of these data to physiological and stressed conditions *in vivo*. In wild-type, 4-week-old female mice (C57BL/6), *Rnf213* proved to be expressed in various tissues, including brain, heart, great vessels, mononuclear cells and spleen. We verified that the heart was the organ with the highest expression of *Rnf213* (Fig. 1f). When we treated these mice with an intra-peritoneal injection of murine IFNG and TNFA, the mRNA level of *Rnf213* was significantly elevated at 6 hr after injection and rapidly declined within 24 hr (Fig. 1g). Intriguingly, the IFNG and TNFA injection activated the expression of *Rnf213* most prominently in heart and great vessels among other tissues. Together, these results demonstrate that *RNF213* is activated by inflammatory signals from the environment both *in vitro* and *in vivo*.

### AKT and PKR pathways up-regulate the transcription of *RNF213*

**Figure 2:**
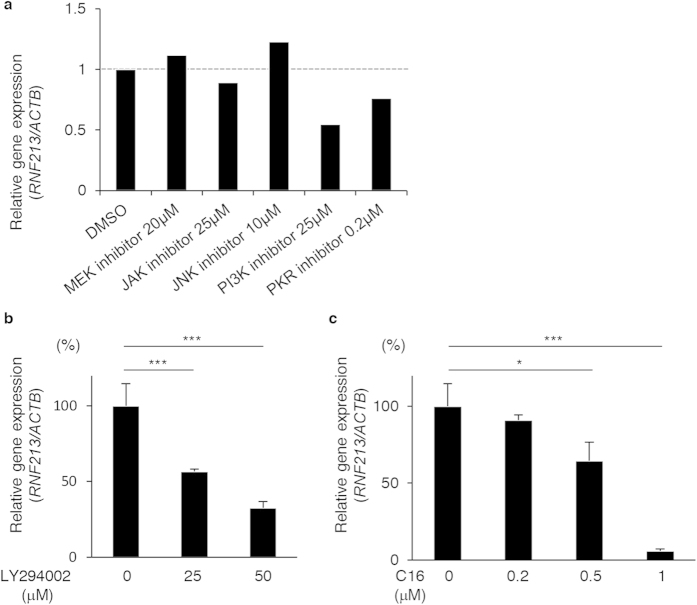
Phosphatidylinositol-4,5-bisphospate 3-kinase and double-stranded RNA-dependent protein kinase are the two upstream regulators of *RNF213* expression. (**a**) Plots show the relative expressions of *RNF213* mRNA in the presence or absence of protein kinase inhibitors for MEK (U0126, 20 μM), JAK (AG490, 25 μM), JNK (SP600125, 10 μM) and PI3K (LY294002, 25 μM) and PKR (C16, 0.2 μM). Mean values from two independent assays are shown. (**b**,**c**) Dose-dependent inhibition of *RNF213* induction by LY294002 (**b**) and C16 (**c**). Relative expressions of *RNF213* are plotted against various concentrations of PI3K and PKR inhibitors. Data are shown as values of mean ± SD from three independent assays and analyzed using Dunnett’s test (**b**,**c**). *p < 0.05, ***p < 0.001.

To identify the upstream pathway(s) that controlled the transcriptional activation of *RNF213* in response to cytokines, we treated HUVECs with various protein kinase inhibitors. These included LY294002 for PI3K-AKT, C16 for PKR, U0126 monoethanolate for MEK-ERK, AG490 for JAK-STAT, and SP600125 for JNK^22^,^23^. Among them, LY294002 and C16 significantly suppressed the transcriptional activation of *RNF213* in endothelial cells upon IFNG treatment (Fig. 2a), and their inhibitory effects were dose-dependent (Fig. 2b,c). These data indicate that PI3K-AKT and PKR are two major upstream regulators of *RNF213* expression in endothelial cells, although it remains to be determined whether other unknown cascades might contribute to the transcriptional activation of *RNF213*.

### RNF213 promotes endothelial cell proliferation

**Figure 3:**
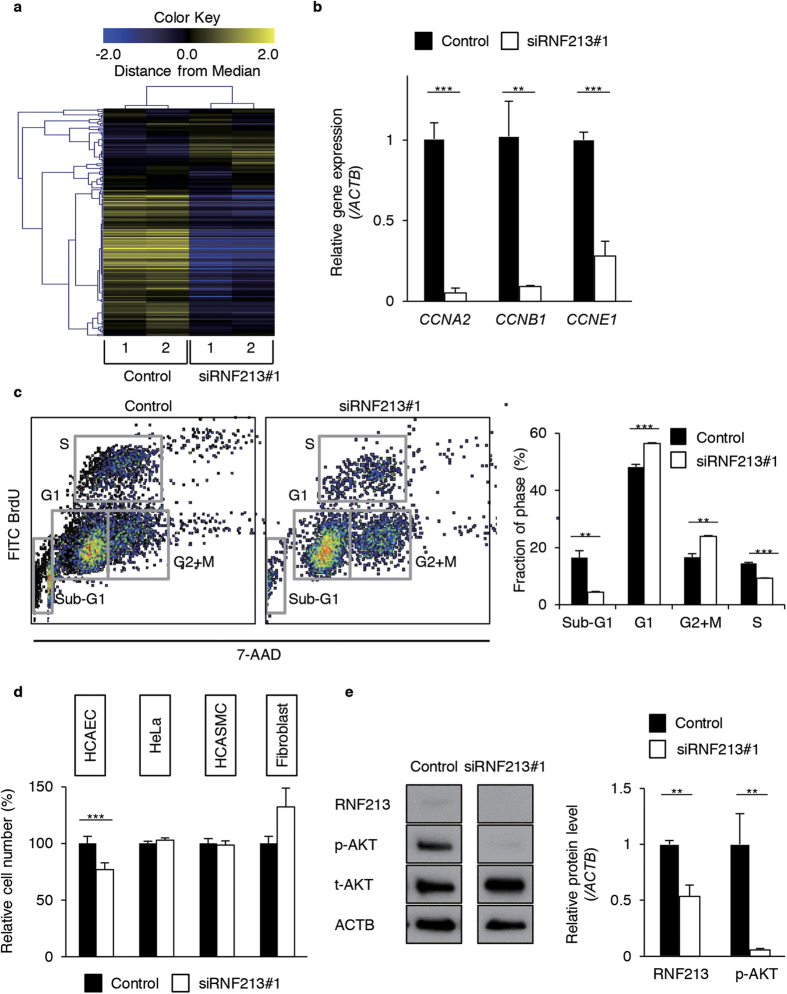
RNF213 up-regulates cell-cycle and proliferation of endothelial cells. (**a**) The heat map shows up (yellow) or down-regulated (blue) genes in siRNF213#1-treated or untreated HCAECs (n = 2 for each condition). Clustering of siRNA-treated cells and expression profiles for each experiment were conducted blindly. (**b**) Validating qPCR assays for *CCNA2*, *CCNB1* and *CCNE1* expressions in HUVECs with or without *RNF213* knockdown (mean ± SD, n = 3, using Student’s t-test). **p < 0.01, ***p < 0.001. (**c**) Flow-cytometry analysis for cell cycles of HUVECs. Used siRNAs (siRNF213#1 or control) are denoted at the top. The left two panels show 2D-plots for fluorescence intensity of FITC-labeled BrdU and that of 7-AAD. Fractions (%) of cells in G0/G1, S, G2+M and sub G0/G1-phases are indicated with squares. Bar plots on the right shows significant decrease in S-phase with siRNA-mediated knockdown of *RNF213* (n = 3, using Student’s t-test, ***p < 0.001). (**d**) MTS assay for HCAECs, HeLa, HCASMCs and fibroblasts in the presence of *RNF213*-specific siRNA (siRNF213#1) or control siRNA (n = 3 in each group, using Student’s t-test, ***P < 0.001). (**e**) Western blots for phosphorylated form of AKT (p-AKT), total AKT (t-AKT) and ACTB in HUVECs. Quantitative data from three independent Western blot analyses are shown as plots on the right (mean ± SD) and analyzed using Student’s t-test. **p < 0.01. Full length blots are presented in Supplementary Fig. S7.

We next investigated the biological impacts of RNF213 depletion in endothelial cells. To identify downstream events, we tested whether siRNA-mediated knockdown of *RNF213* may lead to aberrant expressions of endogenously expressed genes in endothelial cells. Administration of siRNAs to *RNF213* (siRNF213#1 and #2) for 48 hr resulted in profound decrease in RNA (13–46%) and encoded protein (0%), indicating the efficient and rapid degradation of *RNF213* transcripts in the host cells within the time window (Supplementary Fig. S3). We next performed transcriptome analysis of HCAECs upon treatment with siRNF213#1 or with a control siRNA. Overall, a total of 217 genes were up-regulated (>2.0-fold change in expression), while 499 genes were down-regulated (<0.5-fold change in expression), in the siRNF213#1-treated cells when compared to the control (Supplementary Table S3 and Supplementary Table S4). The clustered gene matrix showed that differentially expressed genes between the test and control samples exhibited similar expression profiles within each group, indicating the high-confidence data of our transcriptome analysis (Fig. 3a).

To our surprise, a GO analysis highlighted the overrepresentation of cell cycle-associated genes among those aberrantly expressed in siRNA-treated HCAECs (Supplementary Table S3 and Supplementary Table S4). Specifically, “cell cycle process”, “cell division”, and “DNA replication” were listed among the top 5 GO categories (Supplementary Fig. S4). A KEGG pathway analysis also predicted that such gene expression changes might be linked to deregulation of cell cycle and its associated molecular pathways (Supplementary Fig. S5). Knowing that RNF213 might play an important role for cell-cycle progression in endothelial cells, we carried out the following three experiments to address this issue: First, quantitative (q) PCR assays successfully reproduced the microarray data. The expressions of *CCNA2*, *CCNB1* and *CCNE1* were decreased to 5.1% (p = 0.0002), 9.0% (p = 0.0038) and 28.1% (p = 0.0006) of control, respectively, when we knocked down *RNF213* in HUVECs (Fig. 3b). Second, flow cytometry analyses for BrdU- and 7-AAD-labeled cells revealed that knockdown of *RNF213* caused remarkable decline in the proportion of cells in S-phase (9.3 ± 0.2%), when compared to control cells (14.5 ± 0.4%, p < 0.001, Fig. 3c). In contrast, cells in static phases (G1 and G2+M) were significantly increased. The proportion of cells in Sub-G1 phase was decreased, indicating that apoptotic cells were not increased. Third, the MTS assays showed that *RNF213* knockdown led to a decrease in cell growth to 76.9 ± 5.9% of control in HCAECs (p < 0.001, Fig. 3d). In contrast, cell growths were not disturbed with siRNA treatments of non-endothelial cells, such as HeLa, HCASMCs or fibroblasts (Fig. 3d).

These data collectively provided evidence that RNF213 promotes cell proliferation through regulating its downstream pathways, and that endothelial cells are more susceptible to the functional loss of *RNF213* for cell growth than cells from other tissues. In agreement with these data, knockdown of *RNF213* was shown to decrease phosphorylated AKT (pAKT) in HUVECs and HCAECs, indicating lower activity of PI3K-AKT signal in endothelium. On the other hand, such difference was not observed in non-endothelial cells, HeLa, HCASMCs and fibroblasts (Fig. 3e, Supplementary Fig. S6 and Supplementary Fig. S7).

### RNF213 is an upstream regulator of the matrix metalloproteinases

Cell growth-promoting signals, including the PI3K-AKT pathway, are reportedly associated with the angiogenic potential of endothelial cells^24^. This fact may support the cell-autonomous models of MMD, where functional deficits in endothelial RNF213 may lead to angiopathy as a consequence of persistently low PI3K-AKT activity. However, knowing that the endothelial AKT signals were not activated with the IFNG treatments (Supplementary Fig. S7), we hypothesized that RNF213 might mediate angiogenic responses of endothelial cells through PI3K-AKT-dependent and -independent mechanisms under inflammatory stresses. We therefore inspected minor changes in the microarray data searching for the genes that appeared to be independent of cell cycle and PI3K-AKT pathways.

**Figure 4:**
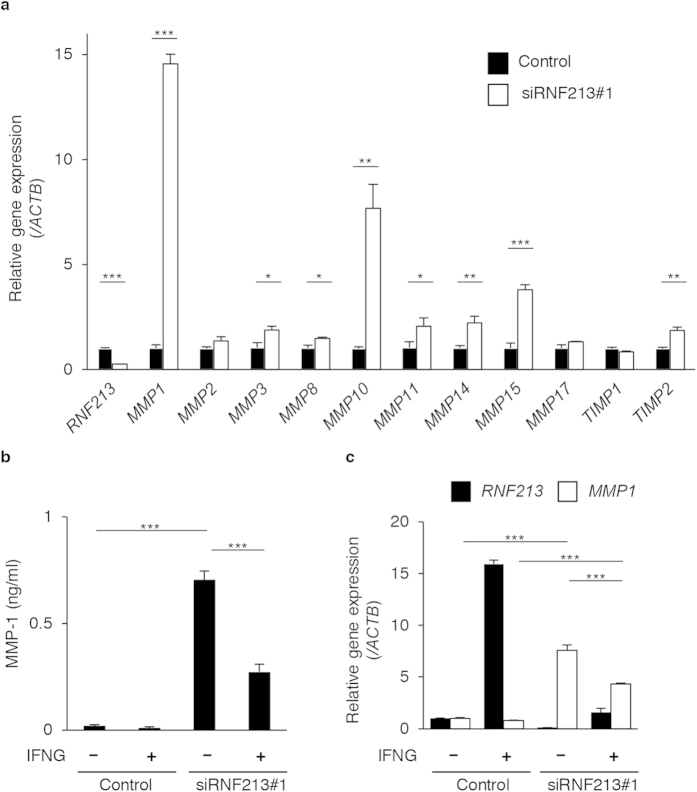
RNF213 controls the expression of MMPs in endothelial cells. (**a**) Relative expressions of *RNF213*, *MMPs*, *TIMP1* and *2* genes are plotted. Note the significant up-regulations of all but *MMP2*, *17*, *TIMP1* genes with knockdown of *RNF213* (white bars) compared to control experiments (black bars). Data are show as values of mean ± SD (n = 3) and analyzed using Student’s t-test. *p < 0.05, **p < 0.01, ***p < 0.001. (**b**) Secreted MMP1 proteins in the culture medium. Data from three independent assays with ELISA are shown (mean ±SD). Note that MMP1 proteins are significantly elevated with knockdown of *RNF213* regardless of IFNG pretreatments. The elevation of MMP1 with *RNF213* knockdown are partly attenuated by IFNG pretreatments (IFNG+) compared to that in untreated cells (IFNG**−**), using Tukey’s HSD test. ***p < 0.001. (**c**) Preventative effects of IFNG on aberrantly up-regulation of *MMP1* RNA due to *RNF213* knockdown. The data from three independent experiments followed by qPCR are shown and analyzed using Tukey’s HSD test. ***p < 0.001.

We found, among aberrantly expressed genes and GO categories, that matrix metalloproteinase (MMP) genes were significantly up-regulated when *RNF213* was knocked down in endothelial cells (Supplementary Table S3). We therefore examined the expression changes of *MMPs* (*MMP1*, *2*, *3*, *8*, *10*, *11*, *14*, *15* and *17*) and of tissue inhibitors of metalloproteinases (*TIMP1* and *TIMP2*) upon knockdown of *RNF213* in HUVECs. A qPCR assays confirmed that all *MMPs* herein tested were elevated following *RNF213* silencing. We primarily focused on MMP1 because *MMP1* was the most prominently up-regulated gene among other *MMPs* with siRNA treatments to *RNF213* (Fig. 4a). This result was confirmed at the protein level when MMP1 protein level in the culture medium was measured by ELISA (Fig. 4b). Noticeably, such increase in *MMP1* expression was attenuated to 38.7% and 57.2% of control at the protein and RNA level, respectively, by pre-treatment with IFNG (p < 0.001, Fig. 4b,c). These results indicated that RNF213 controls the expressions of MMPs as an upstream regulator, and that RNF213 might play a potential role in angiogenesis through these effects on MMPs.

### *RNF213* and *MMP1* expressions in fibroblasts from MMD patients

**Figure 5:**
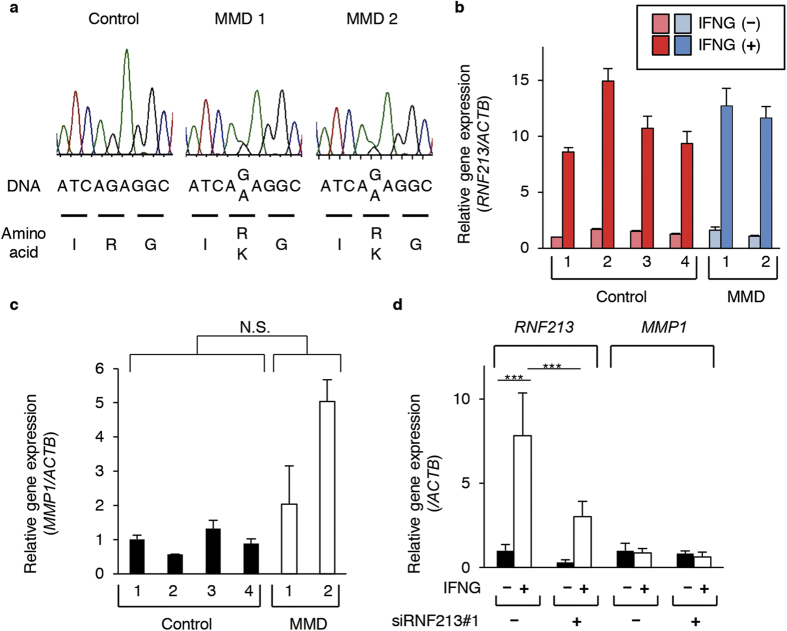
Comparative analysis of *MMP1* expressions in fibroblasts from healthy controls and MMD patients carrying the R4810K variant of *RNF213*. (**a**) Sanger sequences for the target region of *RNF213*. Note that healthy control (Normal) are have the wild-type allele, while two MMD patients (MMD1 and 2) carry a heterozygous c.14756G>A (R4810K) mutations. (**b**) Relative *RNF213* expressions and their responses to IFNG treatment in the fibroblasts from 4 healthy controls (Normal) and 2 MMD patients. Data are shown as mean ± SD values of qPCR assays in three independent assays. (**c**) Relative expressions of *MMP1* transcripts in fibroblasts from 4 individuals of healthy control and 2 MMD patients. Values are shown as mean ± SD (n = 3), and analyzed using Student’s t-test. N.S., not significant. (**d**) Relative *RNF213* and *MMP1* expressions in control fibroblasts with or without treatment by IFNG and siRNF213#1 (mean ± SD, n = 3, using Tukey’s HSD test, ***p < 0.001).

To determine the relevance of above-described results to the pathogenic mechanisms of MMD, we asked whether the variant *RNF213* might have the properties of a hypomorphic allele. We used 4 fibroblasts from healthy volunteers and 2 from MMD patients. The 2 MMD fibroblast lines, but not the 4 controls, were heterozygous with the high-risk allele of *RNF213* (c.14756G>A) (Fig. 5a). In these lines, we confirmed that *RNF213* was similarly induced at mRNA level with IFNG treatments (Fig. 5b). The basal expression levels of *RNF213* as well as its response to the IFNG treatment did not differ between MMD and control groups (Fig. 5b).

We next compared the expressions of *MMP1* mRNA and protein in fibroblasts from MMD patients and healthy individuals. Surprisingly, one of fibroblasts from an MMD patient expressed higher amount of *MMP1* mRNA than controls (p < 0.001), while the other fibroblast did not show significant difference (Fig. 5c). Consequently, we obtained only a marginal difference in the *MMP1* expression between the MMD and control groups (p = 0.052, Fig. 5c). We observed the same trend for MMP1 protein (Supplementary Fig. S8). These data appeared to support that *MMP1* expression varies in individual fibroblasts regardless of the *RNF213* genotypes. Alternatively, however, it might be also possible that the variant *RNF213* allele could affect only minimally the gene expressions in fibroblasts and other non-endothelial cells. In fact, indispensable functions of RNF213 were observed only in endothelial cells (Fig. 4a,b).

We therefore suspected that *RNF213* might function as a dispensable molecule for regulating the *MMP1* expression in non-endothelial cells. To address this issue, we examined whether silencing of *RNF213* in fibroblasts might cause aberrant expressions of *MMP1*. As expected, *MMP1* mRNA expression was not altered with knockdown of *RNF213* in the fibroblasts from a healthy control (Fig. 5d). We further confirmed that IFNG treatment did not result in deregulation of *MMP1* expression in fibroblast in the presence of siRNA for *RNF213* (Fig. 5d). These results were substantially identical in independent assays using fibroblasts from other healthy controls and MMD patients (data not shown). Knockdown of *RNF213* did not alter the expressions of *MMP1* in HeLa or HCASMCs, either (Supplementary Fig. S9).

### Endothelial RNF213 controls angiogenesis through regulating the expression of *MMP1*.

**Figure 6:**
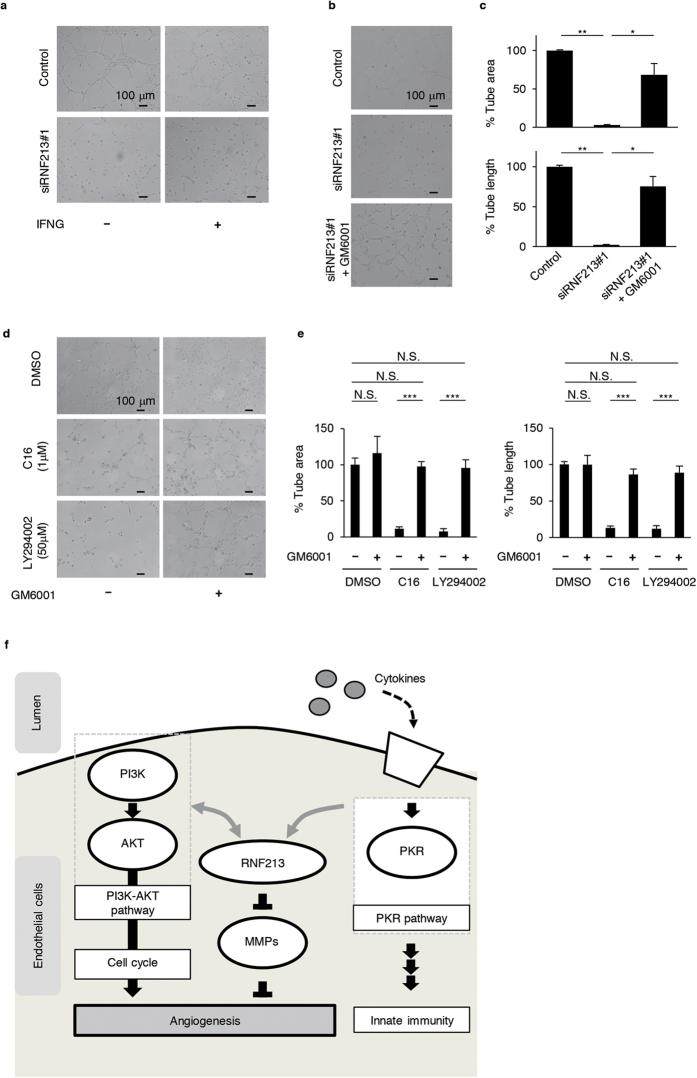
RNF213 links the external signals to angiogenesis through regulating *MMP1* expressions in endothelial cells. (**a**) The angiogenic responses of HUVECs on matrigels in different conditions. Representative images for tubular formation by trypsinized HUVECs in the absence (upper panels) or presence of siRNA for *RNF213* (lower). Effects of IFNG pretreatments (right) on angiogenic response of HUVECs are shown in comparison with those of untreated cells (left). Scale bar = 100 μm. (**b**) MMP is a key downstream molecule for deficits in angiogenic response of endothelial cells with depleted expression of *RNF213*. Upper, middle and lower panels show tubular formation of HUVECs on matrigel without siRNA treatment (“Control”), with siRNA-mediated knockdown of *RNF213* and with co-administration of GM6001, an MMP1 inhibitor, respectively. Scale bar = 100 μm. (**c**) The bar plots show quantitative results of % tube area (upper) and % tube length (lower) on matrigels using HUVECs (n = 3) for Fig. 6b. Tukey’s HSD test. *p < 0.05, **p < 0.01. (**d**) Effects of PI3K and PKR inhibitors for tubular formations of HUVECs on the matrigel and its recovery by GM6001. Scale bar = 100 μm. (**e**) Bar plots presenting quantitative results of % tube area (left) and % tube length (right) on matrigels using HUVECs (n = 3) for Fig. 6d. Tukey’s HSD test. ***p < 0.001. N.S., not significant. (**f**) A proposed model for the regulatory roles of RNF213, PI3K and PKR pathways in endothelial response to cytokines and in angiogenesis.

Although *RNF213* have been shown to be essential for normal vascular development^12^,^25^, it still remains unknown whether the angiogenic functions of RNF213 is associated with inflammatory signals. As previously reported, the matrigel system showed rapid morphological changes of HUVECs and HCAECs into vascular structures within 8 hr after inoculation (Fig. 6a and Supplementary Fig. S10)^26^. Based on the previous data in this study, we predicted that functional loss of RNF213 or its upstream pathways might lead to deficits in such angiogenic responses. Indeed, we found that chemical inhibition of both PI3K-AKT and PKR pathways—the two upstream regulators of RNF213—efficiently disrupted angiogenesis (Supplementary Fig. S11 and Supplementary Fig. S12). Moreover, we found that angiogenic potentials of HUVECs and HCAECs were nearly completely ablated by the siRNA-mediated knockdown of *RNF213* both in presence and absence of IFNG pre-treatments (Fig. 6a and Supplementary Fig. S10).

Lastly, we determined if up-regulated MMPs might contribute to exaggerating the poor angiogenesis of HUVECs when *RNF213* was knocked down. To address this issue, we pretreated the cells for 48 hr with siRNA to knockdown the endogenous *RNF213*, and inoculated them onto the matrigels in the presence or absence of the MMP inhibitor, GM6001. We did find that the MMP inhibitor successfully restored the attenuated angiogenesis of HUVECs due to *RNF213* knockdown (0%) to 68.4% of control (p = 0.02, Fig. 6b,c). Furthermore, we confirmed that disrupted angiogenesis of HUVECs by PKR and PI3K inhibitors were nearly completely restored by GM6001 (p < 0.001, Fig. 6d,e). Therefore, RNF213 promoted angiogenesis of endothelial cells through both cell cycle-dependent and -independent mechanisms. Among cell cycle-independent mechanisms, we identified MMP1 as one of the downstream effectors of RNF213 in endothelial cells for their angiogenic responses.

Taken together, we concluded that *RNF213* was inducible by cytokine-mediated signals in both endothelial and non-endothelial cells. By contrast, the key gene expression changes for angiogenic responses were specific to endothelial cells, but not common with non-endothelial cells.

## Discussion

*RNF213* has been recently identified as an MMD susceptibility gene, but the pathogenic mechanism and the functional implications of the variant allele encoding the R4810K-mutant protein remain unresolved^11^,^12^. In this study, we began by collating the expression profiles of *RNF213* from a massive set of transcriptomic data^16^,^27^. The unbiased, genome-wide approach successfully detected extremely high signals of co-expression profiles for *RNF213* in conjunction with other genes that were previously associated with inflammatory responses, pointing out *RNF213* as a candidate gene that plays a role in pathways such as “innate immune response (GO:0045087)”, “positive regulation of I-kappaB kinase/NF-kappaB cascade (GO:0043123)” and “positive regulation of defense response to virus by host (GO:0002230)”.

We thus explored to validate such bioinformatic predictions through biological experiments: First, we found that acute administrations of TNFA and co-stimulations with other pro-inflammatory cytokines dramatically induced transcription of *RNF213* both *in vivo* and *in vitro*. These data were particularly important in that RNF213 might potentially connect previously known environmental factors of MMD to cell-intrinsic models for the disease onset. Experiments with chemical inhibitors for both PKR and PI3K-AKT pathways efficiently blocked the transcriptional activation of *RNF213* after the cytokine treatment, indicating epistatic regulation of *RNF213* by these pathways. We therefore tested whether *RNF213* and these molecular signals might interplay reciprocally in response to pro-inflammatory cytokines. SiRNA-mediated knockdown of *RNF213* in endothelial cells did not affect PKR or PI3K expression in response to TNFA and IFNG co-stimulations. On the other hand, *RNF213* knockdown led to remarkable decrease in phosphorylated AKT (pAKT) signals, as previously suggested^28^. These data clarified the following two points: 1) RNF213 is a downstream target, and not an upstream regulator, of cytokine-mediated PKR pathway; and 2) RNF213 and PI3K-AKT pathway reciprocally interact with or without cytokine stimulations.

Both PKR and PI3K-AKT pathways are major drivers of new protein synthesis, cell growth and autophagy^29^,^30^. Interestingly, endothelial autophagy is known to be essential for protecting endothelial cells from vascular insults and senescence^31^. In the present study, however, we were unable to obtain experimental data supporting the functional role of RNF213 in vascular autophagy (data not shown). Nonetheless, we anticipate that future experiments using *Rnf213*-knockout or its R4810K knock-in mice will provide robust evidence for these issues. The transcriptomic analysis on cultured endothelial cells in this study disclosed that siRNA-mediated knockdown of endogenous *RNF213* disturbed DNA synthesis and cell proliferation. These data supported an established concept that the cell-cycle progression of endothelial cells is correlated with their angiogenic properties^32^. Similarly, as the PI3K-AKT is a well-known pathway for cell growth, tumorigenesis and cancer-related angiogenesis^30^, it is not surprising that the PI3K inhibitor LY294002 hampered the angiogenic phenotypes of endothelial cells in this study. Notably, we found that the PKR inhibitor also prevented *in vitro* angiogenesis. This finding recapitulated the modifying effects of PKR on angiogenesis through eIF2α phosphorylation^33^. We further found that the PKR inhibitor did not suppress the cell-cycle associated genes (data not shown). Together, it was suggested that RNF213 functions as a common downstream effector of PKR and PI3K-AKT pathways in endothelial angiogenesis through exerting its angiogenic effects through distinct molecular mechanisms in each pathway (Fig. 6f).

Based on an assumption that cell-cycle-independent mechanisms also contributed to the angiogenic defects as a consequence of RNF213 deficiency in endothelium, we closely inspected the minor findings in our microarray data. We found that several matrix metalloproteinase genes, including *MMP1*, *3*, *8*, *10*, *11*, *14*, *and 15*, were significantly increased in their expressions. Excessive MMPs are known to cause epithelial to mesenchymal transition, thereby leading to deleterious effects on endothelial cells in maintenance of vascular structures^34^. The MMP inhibitor, GM6001, restored abnormal phenotypes caused by siRNA-mediated *RNF213* silencing, indicating that the loss of *RNF213* was associated with active vascular remodeling through up-regulation of MMPs. These data indicated that RNF213 promoted angiogenesis through cell-cycle-dependent and independent mechanisms.

The knockdown experiments using fibroblasts did not recapitulate the data for over-expression of *MMP1* or downward regulation of PI3K-AKT that were observed for endothelial cells in this study. This discrepancy can be interpreted by hypothesizing that RNF213 promotes cell proliferation in endothelial cells, but not in other cells or tissues, through positive regulation of the PI3K-AKT pathway. From a more general perspective, these results may reflect differential roles of RNF213 in endothelial cells and other tissues including smooth muscle cells and fibroblasts. This perspective might be coherent with the fact that GO terms for co-expressed genes in the bioinformatic dataset did not necessarily overlap with those of our microarray data using endothelial cells.

In the present study, we also asked whether endothelial cell-autonomous models might fit better to the pathogenic processes of MMD than non-cell-autonomous models^35^,^36^. The elevated expressions of *MMP* mRNAs and proteins with reduced expression of *RNF213* in endothelial cells were likely to support the former, endothelial cell-autonomous model. We were unable to obtain direct evidence for higher expression of *MMP1* in vascular tissues from MMD patients. Alternative experiments applying combined methods of induced pluripotent stem cells with *in vitro* differentiation of endothelial cells will offer more clues for pathogenic responses of the cells from MMD patients to environmental signals. Considering previous studies that associated higher levels of plasma MMPs with increased risk of MMD^37^,^38^ and increased vascular MMP-9 in mice lacking RNF213^39^, we speculate that individuals with the R4810K mutation may have a tendency to produce higher amounts of MMPs from endothelial cells upon systemic inflammation.

A recent study identified *GUCY1A3*, which encodes the α1 subunit of soluble guanylate cyclase (sGC), the major receptor for nitric oxide (NO), as the gene mutated in a syndromic form of MMD^36^. This discovery implicated that alterations of NO-sGC pathway might lead to an abnormal vascular-remodeling process in sensitive vascular areas, such as internal carotid artery bifurcations. We surmised that this concept could be also valid with sporadic, non-syndromic forms of MMD. In line with this concept, it would be reasonable to test whether activated NO synthase under inflammatory stress may require RNF213 to down-regulate the production of MMPs.

One of remaining issues to discuss in this study was how the R4810K variant allele of *RNF213* could affect the biochemical function of RNF213—by a loss of function, gain-of function, or dominant mechanism? Since we were unable to observe differential MMP syntheses in fibroblasts from MMD patients when compared to those in healthy controls, we cannot safely conclude that the R4810K variant of RNF213 results in functional loss of the protein. Nonetheless, markedly elevated MMP production upon silencing of *RNF213* in endothelial cells led to deleterious effects on their angiogenic responses. In this scenario, disease-susceptibility amino acid change (R4810K) is more likely linked to the functional deficiency of RNF213 than its gain of function.

Clinical implications of this study will be further strengthened by analyzing the functional roles of RNF213 in the context of vascular insults by virus and other pathogens. Knowing that the variant allele of *RNF213* appeared more frequently in individuals with syndromic forms of MMD than in the control group (our unpublished data), we assume that functional loss of RNF213 may contribute to the development of MMD even in the presence of other genetic causes or environmental risk factors, such as varicella zoster virus infection. Another aspect of clinical implications may include the potential therapeutic targets for MMD with MMP inhibitors. Also, given the active angiogenesis in malignant tissues and inhibitory effects of some MMPs on cancer proliferation, RNF213 could be considered as a target molecule for future cancer treatments^40^.

In conclusion, our study provides new insight into the convergent functions of RNF213 among various genetic and environmental risk factors for the onset of MMD. We will use mouse models to further explore this issue and identify gene-environment interactions of the two main pathways related to RNF213 (PKR and PI3K-AKT) with vascular inflammation.

## Methods

### Bioinformatic search for co-expressed genes

Expression correlation analysis was performed as previously described^16^,^17^. Briefly, g:Profiler^41^ retrieved a large amount of expression data for the most similarly co-expressed genes in a specified Gene Expression Omnibus (GEO, http://www.ncbi.nlm.nih.gov/geo/) dataset. Among them, expression data involving the four selected gene probes for *RNF213* (Affymetrix probes 225931, 230000, 232155 and 241347, Affymetrix, Santa Clara, CA, USA) was obtained from a total of 106 heterogeneous microarray experiments based on the human Affymetrix HG-U133 Plus 2.0 array. To associate highly correlated genes with specific categories of gene functions, Gene Ontology (GO) DAVID analysis (http://david.abcc.ncifcrf.gov/) were applied, and GO terms with more than a fold enrichment >2 and a P-value < 0.01 were retained (Supplementary Fig. S1 and Supplementary Table S1). Gene symbols and coordinates were used according to the UCSC genome browser hg19 (http://genome.ucsc.edu/). Protein domain information was obtained from Human Protein Reference Database (http://www.hprd.org/)^42^.

### Cell culture

Cells were purchased from ATCC (Manassas, VA, USA) and Coriell Institute (Camden, NJ, USA). Human coronary artery endothelial cells (HCAECs) were cultured in EGM-2MV (Lonza, Basel, Switzerland) containing 10% fetal calf serum (FCS)^43^, and human umbilical vein endothelial cells (HUVECs) were cultured in EGM-2 (Lonza) containing 2% FCS^44^. Human coronary artery smooth muscle cells (HCASMCs) were cultured in SmGM-2 (Lonza) containing 5% FCS. HeLa cells and fibroblasts were cultured in Dulbecco’s Minimal Essential Medium (Wako, Osaka, Japan) containing 10% FCS with 1% penicillin/streptomycin (Wako). HCAECs and HUVECs were assayed at passages 10 and 5, respectively. HCASMCs were assayed at passage 8. Fibroblasts were used at passage between 3 to 5. All cell cultures were maintained at 37 °C in normoxic environments with 5% CO_2_ and 100% humidity.

### Animal studies

Female C57 BL/6N mice at 4 weeks of ages were used for *in vivo* experiments. Briefly, 0.1 ml of PBS or 250 ng/body of TNFA (Sigma-Aldrich, Saint Louis, MO, USA) and 10 μg/body of IFNG (Sigma-Aldrich) were intraperitoneally injected. Animals were euthanized at 0, 6 and 24 hr after injection and RNA was immediately extracted from various tissues after sacrifice on deep anesthesia (RNeasy Micro Kit, Qiagen, Venlo, Netherlands). Total RNA was used for cDNA synthesis followed by quantitative PCR.

### Quantitative real time PCR

Total RNA was extracted using RNeasy Micro Kit (Qiagen) and synthesized complementary DNA using High-Capacity RNA to cDNA Kit (Life Technologies, Carlsbad, CA, USA) according to the manufacturer’s protocol. Quantitative real time PCR (qRT-PCR) was performed using Fast SYBR Green Master Mix and StepOnePlus (Life Technologies). Human *ACTB* or mouse *Actb* was used as internal control gene. The sequences of each gene specific primers were shown in Supplementary Table S2. The PCR conditions were 95 °C (20 seconds), 40 cycles of 95 °C (3 seconds), and 60 °C (30 seconds). Relative gene expression was calculated by ddCt method^43^,^45^.

### Western blotting

Cultured cells were lysed in Laemmli Sample Buffer (Biorad, Hercules, CA, USA). The total protein concentration in cell lysates was determined using Qubit 2.0 Fluorometer (Life Technologies). Equivalent protein amounts from each sample were separated by polyacrylamide gel electrophoresis using 4–15% Mini-PROTEAN TGX Gels (Biorad). Electrophoresed proteins were transfered to PVDF membranes (Trans-Blot Turbo Transfer Pack, Biorad). Blotted membranes were blocked with 5% milk and incubated at 4 °C for overnight with primary antibodies. Following antibodies were used: ACTB (1:10000; Abcam, Cambrige, United Kingdom), RNF213 (1:200; Sigma-Aldrich), AKT (1:1000; Cell Signaling Technology, Danvers, MA, USA), and phospho AKT (1:2000; Cell Signaling Technology). Light-chain specific anti-rabbit or mouse secondary antibodies conjugated to horseradish peroxidase (211-032-171 or 115-035-174, Jackson ImmunoResearch, West Grove, PA, USA) were used to detect the specific protein signals. Chemiluminescence signals (ImmunoStar LD, Wako) were detected using FluorChem FC2 System (ProteinSimple, San Jose, CA, USA). ACTB was used as an internal control.

### RNA interference

Transfection of small interfering RNA (siRNA) was conducted using Lipofectamine RNAiMAX (Life Technologies) according to the manufacturer’s protocol. Commercially available siRNAs were used to knockdown human *RNF213*, which were herein designated as siRNF213#1 and #2 (Stealth RNAi #HSS126645 (sequences: 5′-UUUAACUGGCAUCUGUUUAAGGCCU-3′ and 5′-AGGCCUUAAACAGAUGCCAGUUAAA-3′) and #HSS184009 (sequences: 5′-UGAAGCAGCUGCCUCAACCCAUCUG-3′ and 5′-CAGAUGGGUUGAGGCAGCUGCUUCA-3′), respectively, Life Technologies). Stealth RNAi Negative Control Low GC Duplex (Life Technologies) was used for controls. To check knockdown of gene expression, qRT-PCR and western blotting were carried out as described above.

### Microarray

Microarray-based transcriptome analyses for HCAECs were performed using Sure Print G3 Human GE microarray kit 8×60 k v2 (Agilent Technologies, Santa Clara, CA, USA), and the expression data were processed with GeneSpring GX software (Agilent Technologies) as previously described^43^,^45^. Bioinformatic analyses for clustering^46^, GO and KEGG pathways (http://www.genome.jp/kegg/pathway.html) were conducted with standard protocols as described elsewhere^47^,^48^,^49^. Our transcriptome data have been deposited in NCBI Gene Expression Omnibus under accession code GSE62348.

### Cell proliferation assay

The number of HUVECs was quantitatively analyzed on standard MTS assays using CellTiter 96 AQueous One Solution Cell Proliferation Assay (Promega, Madison, WI, USA). For S-phase specific labeling of growing cells, HUVECs were incubated in the presence of 10 μM BrdU for 2 hours. Cells were trypsinized and labeled with 7-Aminoactinomycin D, and then the proliferating cells in S phase were visualized with FITC BrdU Flow Kit (BD, Franklin Lakes, NJ, USA) using Epics XL (Beckman Coulter, Brea, CA, USA).

### Angiogenic activity

Endothelial tube formation was assessed using Matrigel (BD) following to the manual. HUVECs or HCAECs were plated at 20,000 cells/well on matrigel-coated 24-well culture dishes. Cells were incubated for 4 hours at 37 °C and were allowed to form tube formations. For quantitation, tube area and length were calculated using Image J software (National Institutes of Health, Bethesda, MD, USA) as previously described^25^.

### ELISA and chemicals

Concentration of MMP-1 in the culture supernatant was measured with Quantikine ELISA (R&D Systems, Minneapolis, MN, USA) according to the manufacturer’s protocol. Other chemicals were purchased from Sigma-Aldrich or Wako Pure Chemical Industries.

### Statistical analysis

Results are shown as means ± standard deviation unless otherwise indicated. The statistical significance between groups was assessed by Student’s t-test, Tukey’s HSD test or Dunnett’s test using JMP software (SAS Institute, Cary, NC, USA). The differences were considered significant when P values were less than 0.05.

### Study approval

Ethical issues concerning this study were approved by the institutional review board at Kyushu University (#22-102). All subjects from MMD patients and healthy volunteers were provided with written forms of informed consent prior to this study. All procedures for animal experiments were approved by institutional review boards for animal care at Kyushu University (#A26-232-0). Experiments herein presented were all conducted in a stringent compliance to the institutional guideline.

## Additional Information

**Accession codes:** Our transcriptome data have been deposited in NCBI Gene Expression Omnibus under accession code GSE62348.

**How to cite this article**: Ohkubo, K. *et al.* Moyamoya disease susceptibility gene *RNF213* links inflammatory and angiogenic signals in endothelial cells. *Sci. Rep.* **5**, 13191; doi: 10.1038/srep13191 (2015).

## Supplementary Material

Supplementary Information
