# Supplementary material for: Moyamoya disease susceptibility gene RNF213 links inflammatory and angiogenic signals in endothelial cells
Source: Sci Rep. 2015 Aug 17;5:13191. doi: 10.1038/srep13191 (PMC4538604; doi:10.1038/srep13191)
Supplement: Supplementary Information [file srep13191-s1.pdf]

Supplementary Information for

Moyamoya disease susceptibility gene *RNF213* links inflammatory and angiogenic signals in endothelial cells

Kazuhiro Ohkubo[1,†], Yasunari Sakai[1,*,†], Hirosuke Inoue[1], Satoshi Akamine[1], Yoshito Ishizaki[1], Yuki Matsushita[1], Masafumi Sanefuji[1], Hiroyuki Torisu[1,3], Kenji Ihara[1,2], Marco Sardiello[4] & Toshiro Hara[1]

1. Department of Pediatrics, Graduate School of Medical Sciences, Kyushu University, Fukuoka 812-8582, Japan
2. Department of Pediatrics, Faculty of Medicine, Oita University, Yufu 879-5593, Japan
3. Section of Pediatrics, Department of Medicine, Fukuoka Dental College, Fukuoka 814-0193, Japan
4. Department of Molecular and Human Genetics, Baylor College of Medicine, Jan and Dan Duncan Neurological Research Institute, Texas Children's Hospital, Houston 77030, USA

*Correspondence to: Yasunari Sakai, M.D., Ph.D.

Department of Pediatrics, Graduate School of Medical Sciences,

Kyushu University

3-1-1 Maidashi, Higashi-ku, Fukuoka 812-8582, Japan.

Phone + 81-92-642-5421

Fax +81-92-642-5435

E-mail: ysakai22q13@gmail.com

† These authors contributed equally to this work

This material contains the following items:

- Supplementary Tables S1-4
- Supplementary Figures S1-12 with legends

Supplementary Table S1 | Gene ontology analysis for the genes co-expressed with *RNF213 in silico*.

GO Accession	GO Term	P-Value	Fold Enrichment	Related Genes
GO:0006955	immune response	2.19E-21	7.04	PSMB10, IFIH1, IFITM3, CCR1, OAS3, RSAD2, OAS1, APOBEC3G, IFI44L, OAS2, CXCL11, IFI35, CXCL10, CD97, TAP2, TAP1, DHX58, FYB, GBP5, SP100, BST2, LYN, CFB, SERPING1, SLAMF7, HLA-E, TRIM22, HLA-F, PSMB9, DDX58, OASL, TNFSF10, APOL1, TNFSF13B, GBP4, GBP2, GBP1
GO:0009615	response to virus	1.20E-19	22.89	IFIH1, BST2, RSAD2, APOBEC3G, IFI44, IFI16, STAT1, TRIM22, IFI35, ISG20, STAT2, DDX58, IRF9, PLSCR1, ISG15, IRF7, MX1, EIF2AK2, MX2
GO:0006952	defense response	2.70E-12	5.55	IFIH1, NMI, CCR1, RSAD2, APOBEC3G, CXCL11, CXCL10, CD97, LGALS3BP, TAP2, TAP1, MX1, MX2, DHX58, SP100, LYN, CFB, SERPING1, SLAMF7, IDO1, APOL2, DDX58, APOL3, SIGLEC1, APOL1, IRF7
GO:0006954	inflammatory response	5.88E-06	5.25	NMI, LYN, CFB, CCR1, SERPING1, IDO1, CXCL11, CXCL10, CD97, APOL2, SIGLEC1, APOL3, IRF7
GO:0045087	innate immune response	9.37E-06	8.57	DDX58, IFIH1, APOL1, SP100, CFB, APOBEC3G, SERPING1, SLAMF7, DHX58
GO:0009611	response to wounding	1.70E-04	3.47	NMI, LYN, CFB, CCR1, SERPING1, IDO1, CXCL11, CXCL10, CD97, APOL2, APOL3, PLSCR1, SIGLEC1, IRF7
GO:0002230	positive regulation of defense response to virus by host	5.55E-04	78.80	DDX58, PML, APOBEC3G
GO:0043123	positive regulation of I-kappaB kinase/NF-kappaB cascade	8.22E-04	8.12	TRIM38, APOL3, CFLAR, TNFSF10, BST2, CASP1

Supplementary Table S2 | Oligonucleotide primers for qPCR assays.

Gene	Forward Primer	Reverse Primer
ACTB	5'-CACCCTGAAGTACCCCATCG-3'	5'-TGCCAGATTTTCTCCATGTCG-3'
RNF213	5'-AACAGCTATTCCGTGGATGC-3'	5'-CCAGAGTGGGTATTCCCTTG-3'
LGALS3BP	5'-CATGAGTGTGGATGCTGAGT-3'	5'-CAGCTTGTGGAAGCACTTG-3'
PSMB9	5'-AGAAGTCCACACCGGGACCAC-3'	5'-TGTCAAACACTCGGTTCACCA-3'
TAP1	5'-TGGTCTGTTGACTCCCTTACAC-3'	5'-AAATACCTGTGGCTCTTGTCC-3'
APOBEC3G	5'-CCGAGGACCCGAAGGTTAC-3'	5'-TCCAACAGTGCTGAAATTCG-3'
IFIH	5'-ATGGAAAAAAAAAGCTGCAAAAGA-3'	5'-GTACTTCCTCAAATGTTCTGCACAA-3'
BST2	5'-TTCTCAGTCGCTCCACCT-3'	5'-CACCTGCAACCACACTGT-3'
STAT1	5'-AACGGAGGCGAACCTGACTTCCA-3'	5'-GGCCTGGAGTAATACTTTCCAA-3'
CFB	5'-TGGAAAACCTGGAAGATGTTT-3'	5'-GGTTGCTTGTGGTAATCGGT-3'
TAP2	5'-TACAACACCCGCCATCAG-3'	5'-AGGTCTCTCCGCCAATACAG-3'
ISG15	5'-GGACAAATGCGACGAACCTCT-3'	5'-GGCCTGGAGTAATACTTTCCAA-3'
MX2	5'-CAGCCACCACCAGGAAACA-3'	5'-TTCTGCTCGTACTGGCTGTACAG-3'
TRIM22	5'-GGTTGAGGGGATCGTCAGTA-3'	5'-TTGGAAACAGATTTTGGCTTC-3'
DDX58	5'-GACTGGACGTGGCAAAACAA-3'	5'-TTGAATGCATCCAATATACACTTCTG-3'
IFIT1	5'-GCCATTTTCTTTGCTTCCCCTA-3'	5'-TGCCCTTTTGTAGCCTCCTTG-3'
IRF7	5'-CAGCGTCGGTGGCTACAA-3'	5'-CGCAGCGGAAGTTGGTTTT-3'
IL6	5'-CCACACAGACAGCCACTCAC-3'	5'-AGGTTGTTTTCTGCCAGTGC-3'

Supplementary Table S2 | Oligonucleotide primers for qPCR assays. (continued)

Gene	Forward Primer	Reverse Primer
CCNA2	5’-TCCAAGAGGACCAGGAGAATATCA-3’	5’-TCCTCATGGTAGTCTGGTACTTCA-3’
CCNB1	5’-GAAGATCAACATGGCAGGCG-3’	5’-GCATTTTGGCCTGCAGTTGT-3’
CCNE1	5’-TTCTTGAGCAACACCCTCTTCTGCAGCC -3’	5’-TCGCCATATACCGGTCAAAGAAATCTTGTGCC -3’
MMP1	5’-ATGCTGAAACCCTGAAGGTG-3’	5’-GAGCATCCCCTCCAATACCT-3’
MMP2	5’-GGCCCTGTCACTCCTGAGAT-3’	5’-GGCATCCAGGTTATCGGGGA-3
MMP3	5’-GTCTCTTTCACTCAGCCAAC-3’	5’-ATCAGGATTTCTCCCCTCAG-3’
MMP8	5’-TGATGAAAAAGCCTCGCTG-3’	5’-TGTTGATATCTGCCTCTCCC-3’
MMP10	5’-CATTCCTTGTGCTGTTGTGTC-3’	5’-TGTCTAGCTTCCCTGTCACC-3’
MMP11	5’-AGACACCAATGAGATTGCAC-3’	5’-GCACCTTGGAAGAACCAAATG-3’
MMP14	5’-CGCTACGCCATCCAGGGTCTCAAA -3’	5’-CGGTCATCATCGGGCAGCACAAAA-3’
MMP15	5’-ACAACTATCCCATGCCCATC-3’	5’-ACCTGTCCTCTTGGAAGAAG-3’
MMP17	5’-TCCAGATCGACTTCTCCAAG-3’	5’-CCACATGGCTTAACCCAATG-3’
TIMP1	5’-GGGCTTCACCAAGACCTA-3’	5’-GAAGAAAGATGGGAGTGGG-3’
TIMP2	5’-CCAAAGCGGTCAGTGAGA-3’	5’-TGGTGCCCGTTGATGTTC-3’
mouse Actb	5’-GGCTGTATTCCCCTCCATCG-3’	5’-CCAGTTGGTAACAATGCCATGT-3’
mouse Rnf213	5’-TAAGGATGTCCGCTCCTGGTT-3’	5’-TTGATGGCAGTATACTTGGCA-3’

Supplementary Table S3 | Up-regulated genes with siRNA-mediated knockdown of *RNF213* in HCAECs (top100).

Order of Gene	GeneSymbol	RefSeq Accession	Z score	Order of Gene	GeneSymbol	RefSeq Accession	Z score
1	UBD	NM_006398	6.45	51	TP53I3	NM_004881	3.17
2	SELE	NM_000450	5.83	52	SULF2	NM_018837	3.17
3	HMOX1	NM_002133	5.66	53	BTG2	NM_006763	3.14
4	LCN15	NM_203347	5.64	54	TP53I3	NM_004881	3.13
5	GALNT7	NM_017423	4.98	55	ZNF219	NM_016423	3.12
6	MRC1	NM_002438	4.98	56	RASSF2	NM_014737	3.12
7	IL4I1	NM_172374	4.96	57	SLC6A16	NM_014037	3.11
8	TRAF1	NM_005658	4.92	58	TMEM132A	NM_017870	3.11
9	MT1F	NM_005949	4.90	59	MMP1	NM_002421	3.08
10	ADAM9	NM_003816	4.74	60	CDO1	NM_001801	3.06
11	KALRN	NM_003947	4.61	61	FAM104A	NM_032837	3.01
12	BIRC3	NM_001165	4.47	62	NRP1	NM_003873	3.01
13	IL8	NM_000584	4.32	63	PRCP	NM_199418	3.01
14	KITLG	NM_000899	4.24	64	FBLL1	NR_024356	3.00
15	VWF	NM_000552	4.05	65	CLDN5	NM_001130861	2.99
16	ZMAT3	NM_022470	4.00	66	CH25H	NM_003956	2.98
17	PLA2G4C	NM_003706	3.95	67	NID1	NM_002508	2.96
18	PSG8	NM_001130167	3.94	68	SNN	NM_003498	2.95
19	LDHB	NM_002300	3.93	69	IL32	NM_001012633	2.89
20	LTB	NM_002341	3.90	70	HIST1H4K	NM_003541	2.87
21	FAS	NM_000043	3.83	71	NEURL3	NR_026875	2.86
22	TSPAN11	NM_001080509	3.76	72	RNF13	NM_007282	2.83
23	VWCE	NM_152718	3.76	73	COL1A2	NM_000089	2.83
24	NUAK2	NM_030952	3.74	74	SLC40A1	NM_014585	2.83
25	SLC7A7	NM_001126106	3.71	75	TMEM217	NM_001162900	2.83
26	CLNS1A	NM_001293	3.71	76	CTSK	NM_000396	2.82
27	ARL6IP1	NM_015161	3.69	77	ICAM1	NM_000201	2.82
28	CXorf36	NM_024689	3.66	78	RAG1	NM_000448	2.81
29	FAS	NM_000043	3.65	79	LRRC8B	NM_015350	2.81
30	MT1M	NM_176870	3.64	80	ID2	NM_002166	2.81
31	FAS	NM_000043	3.57	81	USP46	NM_022832	2.79
32	CEACAM1	NM_001712	3.54	82	CLN8	NM_018941	2.79
33	MMP10	NM_002425	3.51	83	GPIHBP1	NM_178172	2.76
34	PSG2	NM_031246	3.51	84	KLHDC9	NM_001007255	2.74
35	FAM49B	NM_016623	3.50	85	NUPR1	NM_001042483	2.74
36	NID2	NM_007361	3.48	86	SEMA6C	NM_001178061	2.74
37	CSF2	NM_000758	3.43	87	LOC282997	NR_026932	2.71
38	SLC2A3	NM_006931	3.43	88	ID2	NM_002166	2.71
39	PLTP	NM_006227	3.43	89	GPR116	NM_001098518	2.69
40	MT1E	NM_175617	3.42	90	C9orf80	NM_021218	2.69
41	MAP2	NM_002374	3.41	91	LRRC8B	NM_015350	2.69
42	ICK	NM_016513	3.38	92	RPL22	NM_000983	2.67
43	C6orf192	NM_052831	3.37	93	ATL1	NM_181598	2.67
44	PSG8	NM_182707	3.33	94	ACP5	NM_001611	2.63
45	CCL2	NM_002982	3.27	95	C20orf108	NM_080821	2.61
46	IL32	NM_001012631	3.26	96	RAB8B	NM_016530	2.60
47	LDHB	NM_001174097	3.25	97	GPR116	NM_001098518	2.53
48	C7orf41	NM_152793	3.21	98	SIRPB2	NM_001122962	2.52
49	GPR116	NM_001098518	3.20	99	RELB	NM_006509	2.48
50	FZD4	NM_012193	3.19	100	GDF15	NM_004864	2.40

MMPs were highlighted in red.

Supplementary Table S4 | Down-regulated genes with siRNA-mediated knockdown of *RNF213* in HCAECs (top100).

Order of Gene	GeneSymbol	RefSeq Accession	Z score	Order of Gene	GeneSymbol	RefSeq Accession	Z score
1	E2F2	NM_004091	-5.96	51	CDCA8	NM_018101	-4.49
2	FAM167A	NM_053279	-5.80	52	CDC20	NM_001255	-4.49
3	CENPA	NM_001809	-5.80	53	PLK1	NM_005030	-4.48
4	ITGB4	NM_000213	-5.77	54	NUF2	NM_145697	-4.48
5	MKI67	NM_002417	-5.49	55	ANLN	NM_018685	-4.47
6	TOP2A	NM_001067	-5.44	56	CCNA2	NM_001237	-4.47
7	MKI67	NM_002417	-5.38	57	CDKN3	NM_005192	-4.46
8	RPL22L1	NM_001099645	-5.38	58	CDCA5	NM_080668	-4.45
9	KIFC1	NM_002263	-5.37	59	TROAP	NM_005480	-4.43
10	UBE2C	NM_181803	-5.36	60	DIAPH3	NM_030932	-4.42
11	KIF20A	NM_005733	-5.34	61	KIF23	NM_138555	-4.40
12	RRM2	NM_001034	-5.29	62	MKI67	NM_002417	-4.40
13	PRC1	NM_003981	-5.24	63	FAM64A	NM_001195228	-4.39
14	MSMP	NM_001044264	-5.17	64	ASF1B	NM_018154	-4.38
15	H2AFV	NM_012412	-5.17	65	MND1	NM_032117	-4.38
16	RAD54L	NM_003579	-5.11	66	EGR1	NM_001964	-4.38
17	KIF15	NM_020242	-5.08	67	SGOL1	NM_001012409	-4.36
18	NTSR1	NM_002531	-4.99	68	BUB1B	NM_001211	-4.35
19	ASPM	NM_018136	-4.96	69	CDCA7L	NM_018719	-4.32
20	CDC25C	NM_001790	-4.96	70	TGM2	NM_198951	-4.32
21	CENPM	NM_024053	-4.92	71	PTTG1	NM_004219	-4.31
22	KIF2C	NM_006845	-4.90	72	CDKN3	NM_005192	-4.29
23	XRCC6	NM_001469	-4.89	73	CCNB1	NM_031966	-4.29
24	ASPM	NM_018136	-4.87	74	CDK1	NM_001786	-4.29
25	SPC25	NM_020675	-4.81	75	DKK2	NM_014421	-4.24
26	IQGAP3	NM_178229	-4.80	76	SKA1	NM_001039535	-4.23
27	TTK	NM_003318	-4.77	77	CEP55	NM_018131	-4.21
28	PKMYT1	NM_182687	-4.76	78	CASC5	NM_170589	-4.20
29	CIT	NM_007174	-4.71	79	CCNB2	NM_004701	-4.20
30	BUB1	NM_004336	-4.71	80	HIST2H3A	NM_001005464	-4.20
31	CENPM	NM_001002876	-4.70	81	HJURP	NM_018410	-4.20
32	GTSE1	NM_016426	-4.70	82	CKS2	NM_001827	-4.19
33	C9orf140	NM_178448	-4.67	83	DEPDC1B	NM_018369	-4.19
34	DLGAP5	NM_014750	-4.66	84	NUSAP1	NM_016359	-4.13
35	CENPF	NM_016343	-4.66	85	TSPAN8	NM_004616	-4.13
36	CEP55	NM_018131	-4.64	86	NEK2	NM_002497	-4.11
37	PBK	NM_018492	-4.62	87	NCEH1	NM_020792	-4.10
38	VAMP3	NM_004781	-4.61	88	TAF9B	NM_015975	-4.08
39	AURKB	NM_004217	-4.60	89	KIF20B	NM_016195	-4.07
40	ATAD2	NM_014109	-4.59	90	KIF23	NM_138555	-4.06
41	TFRC	NM_003234	-4.58	91	ADAMTS1	NM_006988	-4.05
42	TNFRSF6B	NM_003823	-4.58	92	MLF1IP	NM_024629	-4.01
43	TPX2	NM_012112	-4.58	93	HIST1H1B	NM_005322	-3.99
44	KIF4A	NM_012310	-4.57	94	BCL2A1	NM_004049	-3.97
45	KIF11	NM_004523	-4.57	95	ST6GALNAC1	NM_018414	-3.92
46	DIAPH3	NM_001042517	-4.54	96	CCL23	NM_005064	-3.92
47	RN5-8S1	NR_003285	-4.53	97	RFK	NM_018339	-3.87
48	APOBEC3B	NM_004900	-4.53	98	BRIP1	NM_032043	-3.82
49	NDC80	NM_006101	-4.51	99	HSD11B1	NM_181755	-3.72
50	BIRC5	NM_001012271	-4.51	100	HIST1H3B	NM_003537	-3.35

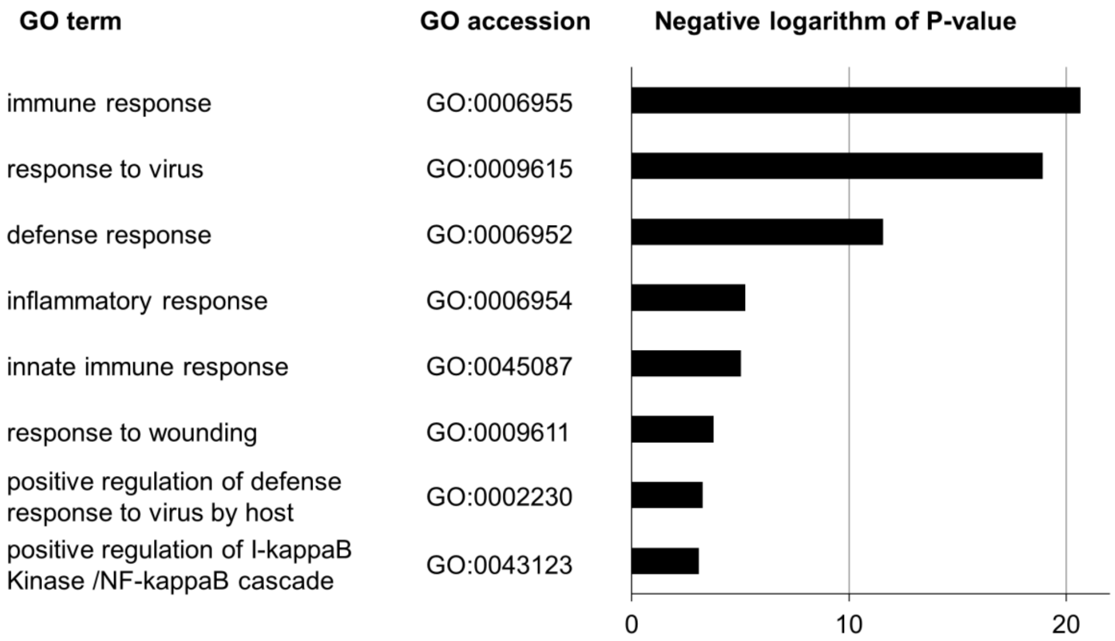

Supplementary Fig. S1 | Negative logarithm of P-value for enrichment of the functional category in gene ontology (GO). The corresponding GO terms and accession codes are listed on the left.

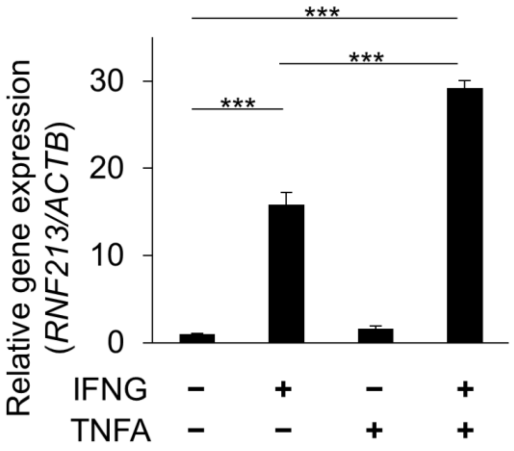

Supplementary Fig. S2 | Synergistic effects of IFNG and TNFA on transcriptional activation of *RNF213* in HCAECs. Relative expression levels of *RNF213* in the presence (+) or absence (-) of IFNG and TNFA are shown as mean ± SD values (n = 3). *ACTB* was used as internal control. ***p < 0.001.

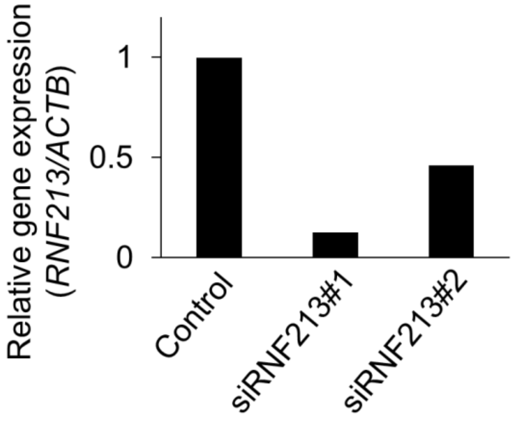

Supplementary Fig. S3 | *In vitro* knockdown of *RNF213* with synthetic siRNA duplexes in HCAECs. Data represent the mean values of relative expression of *RNF213* at 48 hr after treatment of HCAECs with indicated siRNAs. Error bars were omitted because data with >95% of accuracy was obtained in two independent assays.

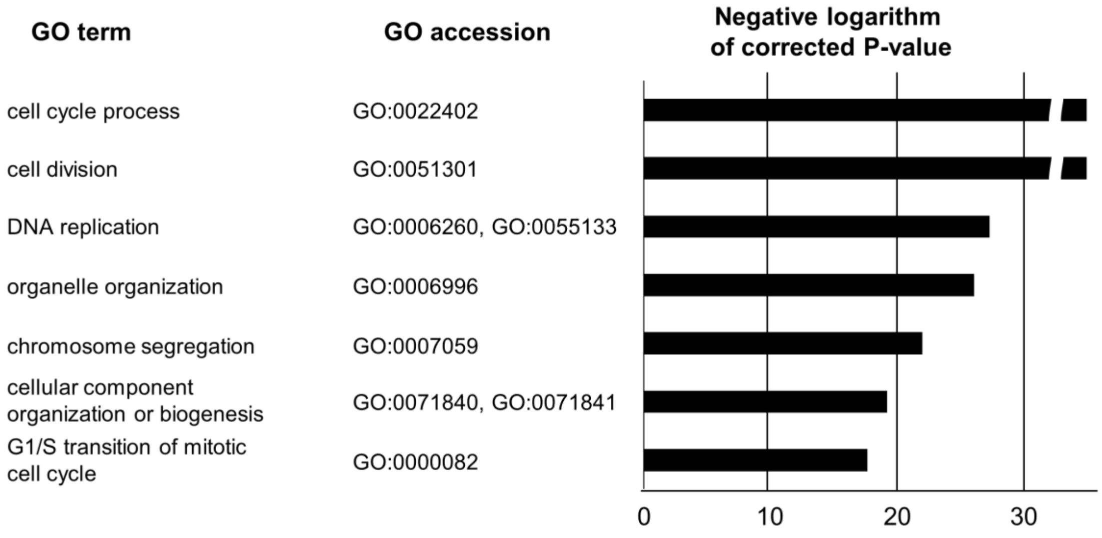

Supplementary Fig. S4 | Negative logarithm of corrected P-value for enrichment of the functional category in gene ontology (GO). The corresponding GO terms and accession codes are listed on the left.

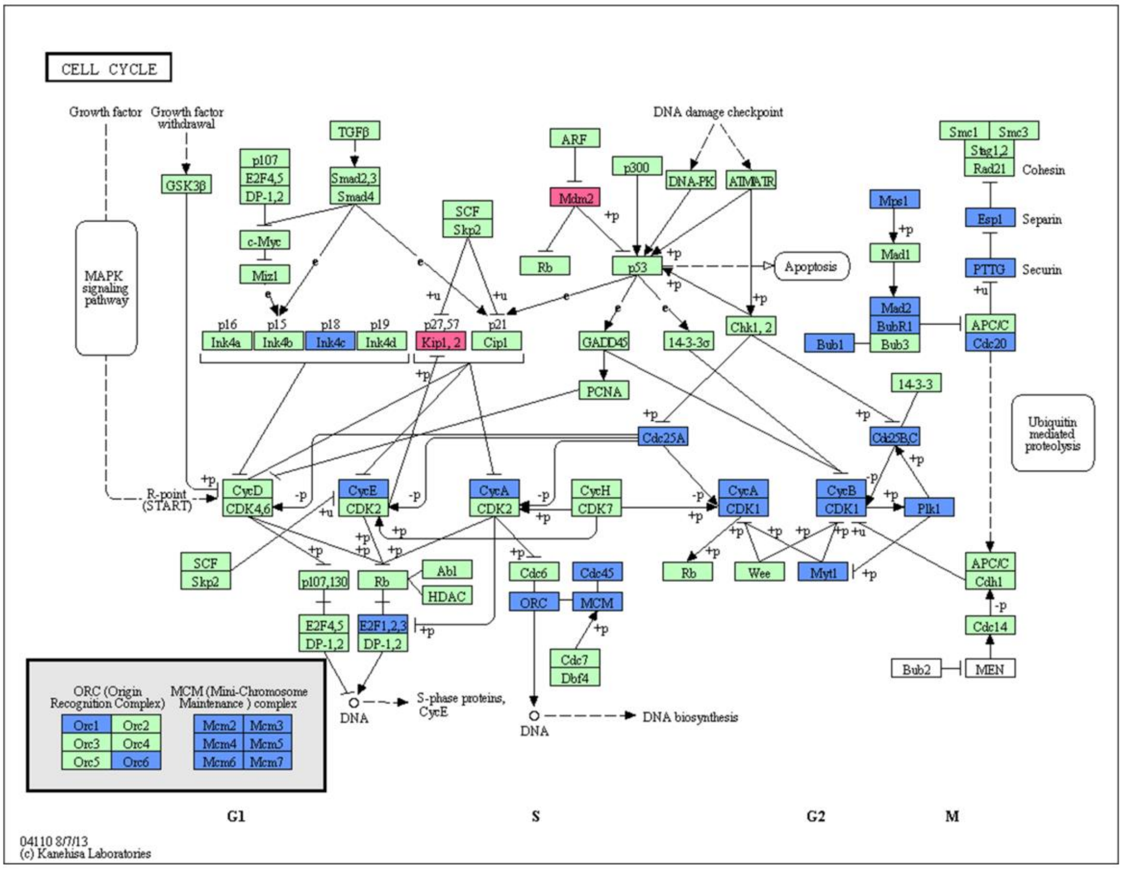

Supplementary Fig. S5 | KEGG pathway analysis for the transcriptome data with siRNA-mediated knockdown of *RNF213* in endothelial cells. The color codes indicate up-regulated (red), down-regulated (blue) and other genes (green).

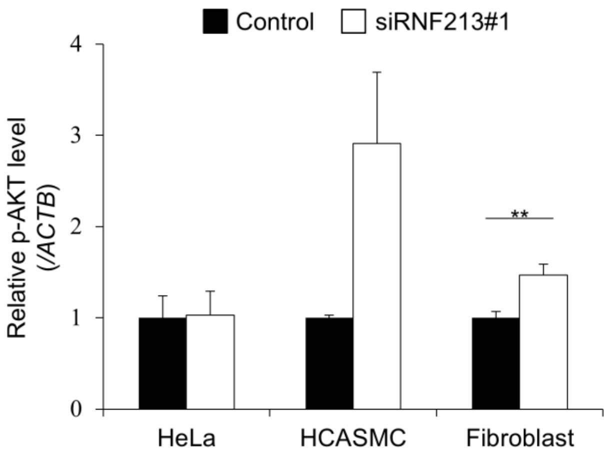

Supplementary Fig. S6 | Western blots for p-AKT level for HeLa, HCASMCs or fibroblasts. SiRNA-mediated knockdown of *RNF213* (siRNF213#1) did not decrease p-AKT level in those cells (n = 3 in each group). "Control" represents the cells treated with control siRNA. Data are shown as mean ± SD values and analyzed using Student's t-test. **p < 0.01.

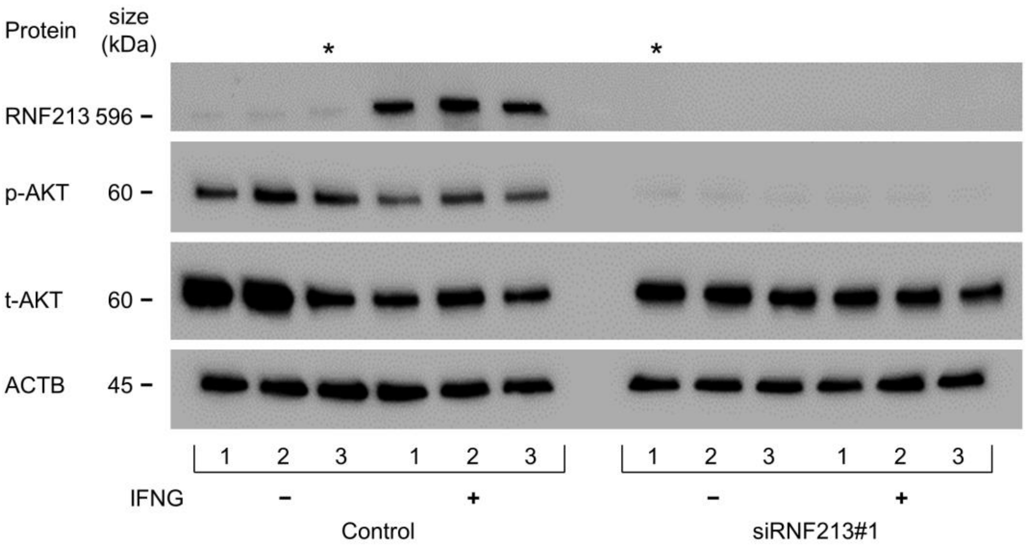

Supplementary Fig. S7 | The original western blot images in HUVECs. The immuno-probed proteins and the molecular size are indicated on the left. The footnotes annotate the conditions of IFNG and the siRNA treatments in triplicate (1-3). Asterisks indicate the two lanes selected for **Fig. 3e**.

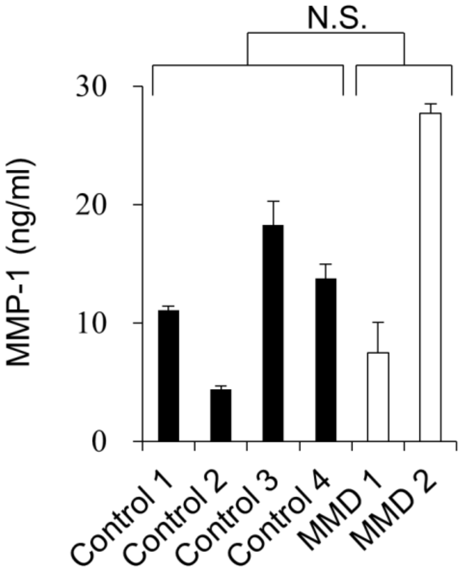

Supplementary Fig. S8 | MMP1 protein levels at basal condition in fibroblasts from 4 healthy controls and 2 MMD patients. Data are shown as mean ± SD values (n = 3) and analyzed using Student's t-test. N.S., not significant.

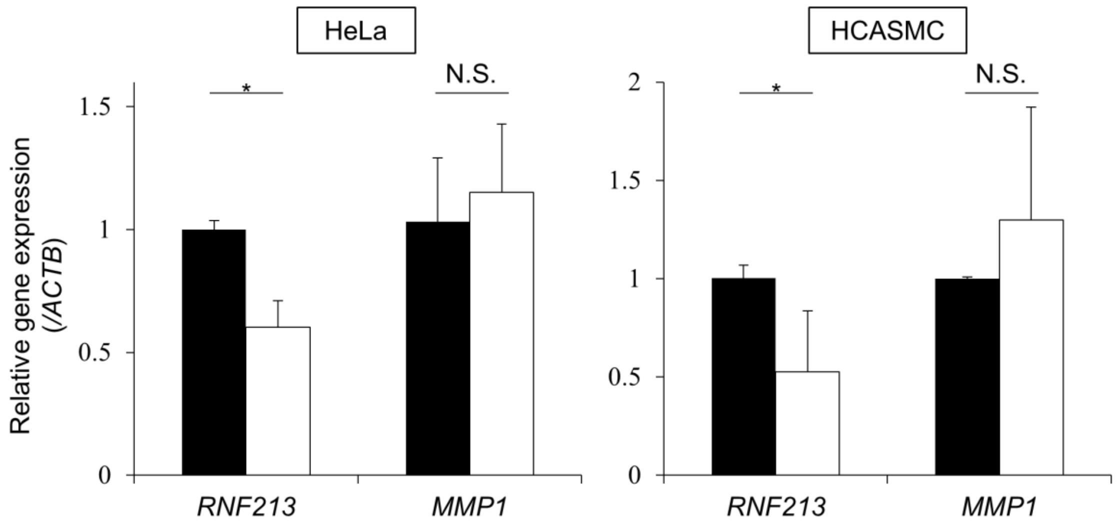

Supplementary Fig. S9 | Negligible effects of the *RNF213* silencing on *MMP1* expression in HeLa and HCASMAs cells. Plots are shown as mean ± SD (n = 3) and analyzed using Student's t-test. *$p < 0.05$. N.S., not significant.

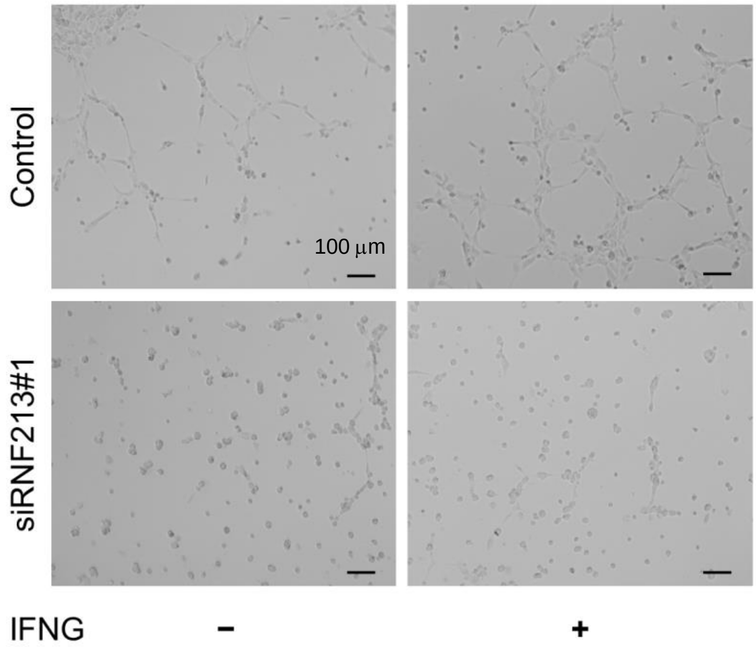

Supplementary Fig. S10 | The angiogenic responses of HCAECs on matrigels in different conditions. Representative images for tubular formation by trypsinized HCAECs in the absence (upper panels) or the presence of siRNA for *RNF213* (lower). Effects of IFNG pretreatments (right) on angiogenic response of HCAECs are shown in comparison with those of untreated cells (left). Scale bar = 100 μm.

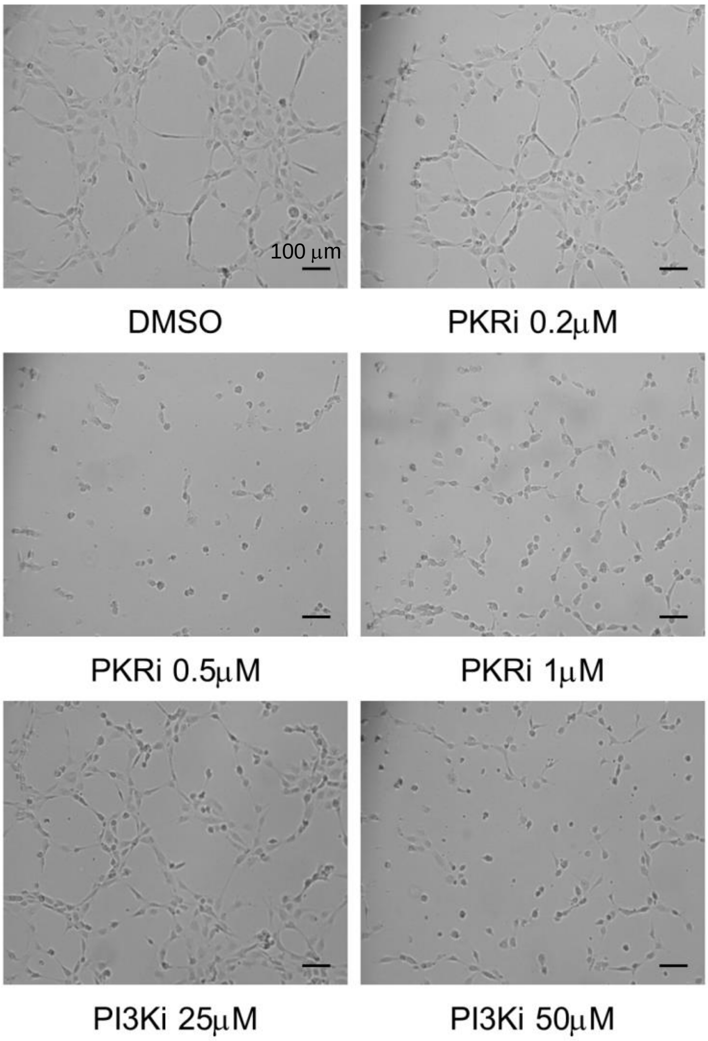

Supplementary Fig. S11 | PI3K and PKR inhibitors disrupt the tubular formations of HUVECs on the matrigel. The images of growing HUVECs on matrigels were captured at 4 hr after inoculation. Applied compounds (DMSO, LY294002 and C16) are annotated at the bottom of each panel. Scale bar = 100 μm.

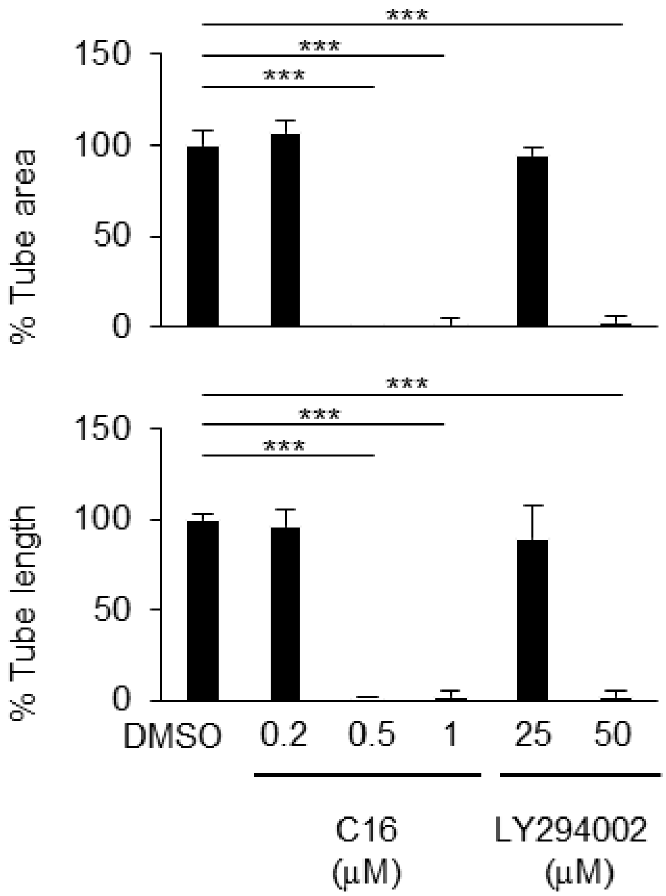

Supplementary Fig. S12 | The quantitative data for Supplementary Fig. S11. % tube area (upper) and length (lower) are shown as mean ± SD plots (n = 3) and analyzed using Dunnett's test. ***p < 0.001.
